# A Model of the Ventral Visual System Based on Temporal Stability and Local Memory

**DOI:** 10.1371/journal.pbio.0040120

**Published:** 2006-04-18

**Authors:** Reto Wyss, Peter König, Paul F. M. J Verschure

**Affiliations:** **1**Institute of Neuroinformatics, University/ETH Zürich, Zürich, Switzerland; **2**Institute of Cognitive Science, University Osnabrück, Neurobiopsychologie, Osnabrück, Germany; **3**Computation and Neural Systems, California Institute of Technology, Division of Biology, Pasadena, California, United States of America; **4**ICREA and Technology Department, University Pompeu Fabra, Barcelona, Spain; Columbia UniversityUnited States of America

## Abstract

The cerebral cortex is a remarkably homogeneous structure suggesting a rather generic computational machinery. Indeed, under a variety of conditions, functions attributed to specialized areas can be supported by other regions. However, a host of studies have laid out an ever more detailed map of functional cortical areas. This leaves us with the puzzle of whether different cortical areas are intrinsically specialized, or whether they differ mostly by their position in the processing hierarchy and their inputs but apply the same computational principles. Here we show that the computational principle of optimal stability of sensory representations combined with local memory gives rise to a hierarchy of processing stages resembling the ventral visual pathway when it is exposed to continuous natural stimuli. Early processing stages show receptive fields similar to those observed in the primary visual cortex. Subsequent stages are selective for increasingly complex configurations of local features, as observed in higher visual areas. The last stage of the model displays place fields as observed in entorhinal cortex and hippocampus. The results suggest that functionally heterogeneous cortical areas can be generated by only a few computational principles and highlight the importance of the variability of the input signals in forming functional specialization.

## Abstract 2

A robot-controlled camera feeds natural stimuli into an unsupervised computational model that constructs a hierarchy of processing stages resembling the ventral visual pathway, from primary visual cortex up to entorhinal cortex.

## Introduction

The processing of visual information is a fundamental computational task for the brain involving various cortical and subcortical regions. Starting at the retina and thalamus, visual information passes through a series of hierarchically organized cortical regions eventually reaching higher cognitive structures such as the hippocampus [ 1]. Experimental studies have shown that the different levels of the ventral visual hierarchy form increasingly complex and specific representations of the visual input such as three-dimensional objects and faces in the inferotemporal cortex (IT) [ 2– 5] and an allocentric representation of space in entorhinal cortex [ 6] and hippocampus [ 7]. This process is accompanied by an increasing degree of invariance to various stimulus properties [ 8]. Thus, the ventral visual stream presents itself as a hierarchical system with widely varying properties at different processing levels.

In recent years, different models of the ventral visual system have been proposed that aim to account for these properties [ 9– 13]. Although most of these theoretical studies emphasize the important role of learning for the adaptation of a visual system, it is often limited to certain stages of processing only or performed on artificial stimuli, both with respect to their temporal and spatial properties. Furthermore, none of these models considers levels of the visual hierarchy as high as the entorhinal cortex or hippocampus. In a complementary line of research, theoretical studies have shown that prominent computational properties of the primary visual cortex can be described by means of so-called objective functions. Important examples are optimally sparse representations resembling simple cells [ 14– 16] and optimally stable representations giving rise to complex cells [ 11, 17– 19]. It remains unresolved, however, whether objective functions can describe general principles underlying cortical organization. Here we show that the objective of optimal stability of sensory representations combined with local memory can generate a hierarchy of cortical-like processing stages resembling the ventral visual pathway. This model visual hierarchy is generated on the basis of visual stimuli encountered by a mobile robot exploring a complex real-world environment. We show that the receptive fields at the lowest level of the hierarchy share properties with those observed in the primary visual cortex, that higher levels are selective for complex configurations, as observed in the IT, and that the last stage of the model ventral visual system displays place fields as observed in entorhinal cortex and hippocampus [ 7]. These results suggest that a substantial part of the visual system can be understood based on a small number of principles.

## Results

**Figure 1:**
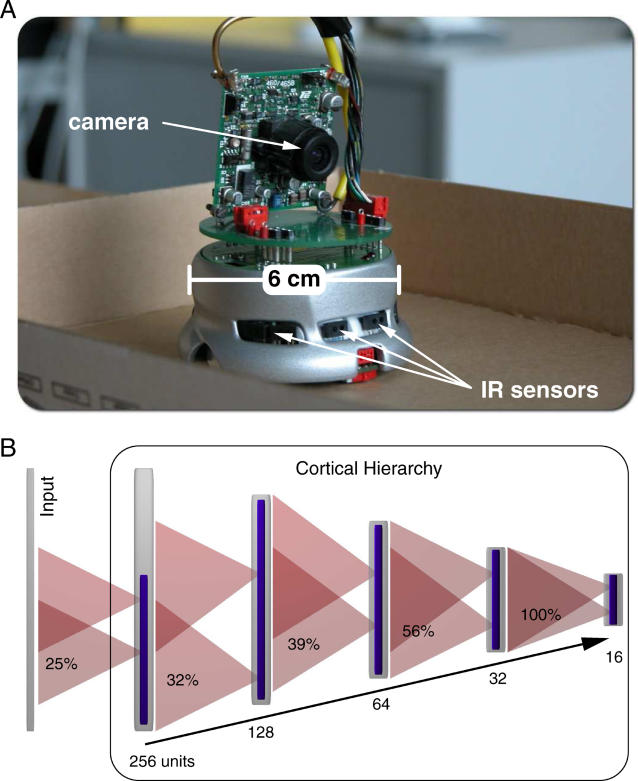
The Micro-Robot Khepera and the Neural Network Structure Used for Sensory Processing (A) A camera mounted on top of the cylindrical body provides the visual input that is processed by our model of the ventral visual system. The infra-red (IR) sensors are used for obstacle avoidance during exploration of a real-world office environment within an arena of approximately 31 × 22 cm ^2^. (B) Diagram showing the hierarchical network comprising five levels of identical computational units. Units are arranged uniformly within a two-dimensional square lattice, and their number per level decreases with a constant factor of 0.5 moving up the hierarchy. Each efferent unit receives input from a topographically aligned square region within the afferent level (red connectivity) and connects laterally to all the units in the same level with which it shares feed-forward input (blue connectivity). The average relative size of a unit's feed-forward arbor within the afferent level (as given in percentages), and consequently also the lateral degree of connectivity, increases with the hierarchical level and reaches 100% for the units at the highest level. The input to the network has a resolution of 16 × 16 pixels.

We investigate the adaptation and specialization of areas in a hierarchically organized visual processing stream using both a real-world robot, as well as a simulated virtual approximation (see Materials and Methods). The agents are embedded in a complex environment, and a camera mounted on the robot provides continuous input to the neural network ( Figure 1A). The model of the visual system consists of five areas each comprising units with both intra-area and feed-forward inter-area connections ( Figure 1B). The convergence of the feed-forward connectivity increases while moving up the hierarchy, similar to that observed in the visual pathway [ 1]. These feed-forward connections are subject to online unsupervised learning optimizing a temporal stability objective while the intra-area connections serve the decorrelation of the states of one area. In addition, all units are leaky integrators providing them with a local transient memory trace. The data analysis focuses on the learning and network dynamics and a comparison of the response properties of neurons at different levels of the hierarchy with their respective counterparts in the real brain.

**Figure 2:**
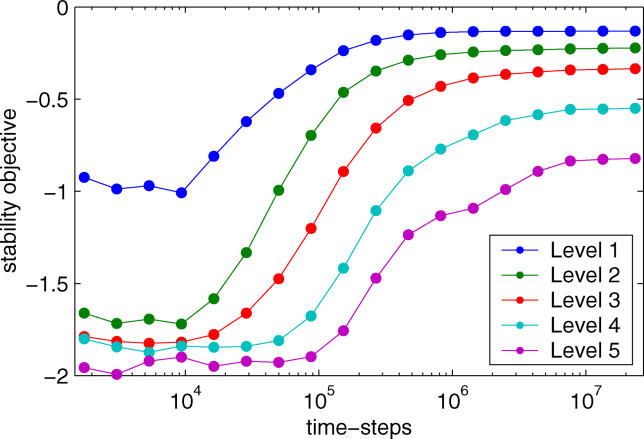
The Stability Objective as a Function of Time for the Five Different Cortical Levels After an initial phase, within which the transients due to initial conditions have decayed, learning is initiated after 10,000 time steps.

**Figure 3:**
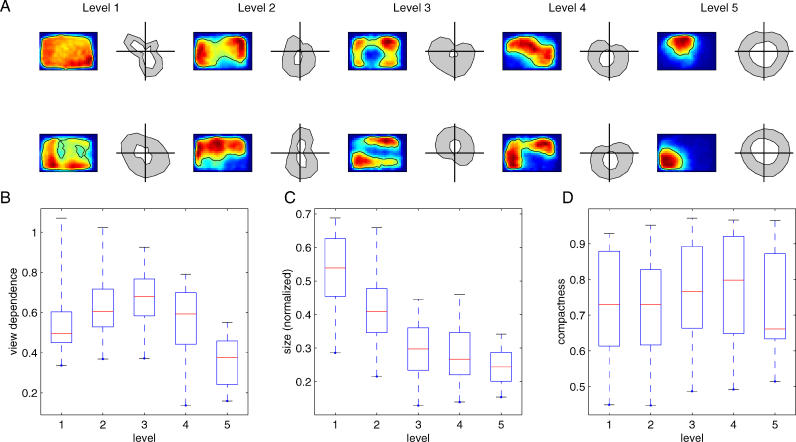
Response Properties of Units with Respect to Behavioral Space (A) Responses of two-example units per hierarchical level with respect to the robot's position (left column) and its orientation (right column). The response maps show responses of cells averaged across the robot's orientation where the black contour (responsive region) identifies the 50% level of the maximal response. The polar plot shows the mean ±SD of the unit's activity within the black region with respect to 16 equally spaced orientations covering 360°. Both response maps and polar plots are normalized to the maximal response of the units across space or orientation, respectively. (B and C) Boxplots of the view, dependence, size, and compactness of the responsive regions versus the hierarchical level of the units. The blue box represents the upper and lower quartiles. The median is indicated by the red horizontal line whereas the extent of the remaining data is given by the vertical whiskers. The view dependence is measured as the CV of the response of a unit across orientations for a fixed position, averaged across the responsive region. The size of the responsive region is normalized to the size of the environment. The compactness is given by the ratio between the true perimeter of the 50% contour and the perimeter of a disc with equal area.

Exposing the network to the visual input provided by the mobile robot, we observe that after approximately 6 ·10 ^6^ time steps (66 h of real-time, with a frame rate of 25 Hz) the stability of all levels has converged ( Figure 2). In addition, the model shows that the reorganization of the levels, in terms of their stability, follows the hierarchical order of the system, i.e., higher levels enhance their stability only after their afferent levels have reached a certain level of stable representations. After convergence, units at different processing levels show characteristic differences in their response properties. In particular, cells at the first level show orientation selectivity confirming previous results [ 19, 20] (unpublished data). In the following, we analyze the response properties of the cells at different levels with respect to the orientation and position of the robot within the environment ( Figure 3A). Both the view dependence and the size of the region where activity can be elicited varies significantly with respect to the hierarchical level (one-way ANOVA, F(4,491) = 30.6, 128.3, respectively, *p* ≪ .001). The view dependence of the units increases from the first to the third level on average by 16% and subsequently decreases and reaches its minimum at the last level, 32% below the first level ( Figure 3B). In contrast, the size of the regions in which individual units are activated decreases monotonically ( Figure 3C). On average, units at the first level are responsive within 52% of the environment. In contrast, units at the highest level only cover 24% of the environment. In order to control for an increasing fragmentation of the representations, we also measure the compactness of the responsive region, which does not change significantly across the hierarchical levels ( Figure 3D, F(4,491) = 1.28, p > 0.2). In summary, these results show that our model captures pertinent properties of the ventral visual stream. The response properties of units at early stages are selective to low-level features. Such features are visible from many different positions within the environment and the responsive regions tend to be large and selective for the orientation of the robot. At intermediate stages, each unit responds specifically to a particular view from a region of limited size, similar to landmarks, leading to a high orientation selectivity. Higher levels learn to associate neighboring “landmark” views, rendering small, compact responsive regions. At the highest level, these “landmark” representations are combined into an allocentric representation of space: a place field that is highly selectively for the robot being at a certain position within the environment irrespective of its orientation [ 7, 21].

The receptive field (RF) sizes of the feed-forward projections are bounded by the synaptic arbors of the cells at the different levels of the hierarchy. Optimizing the weights of these synapses, however, may result in different effective RF sizes. Therefore, we subsequently compare the optimized hierarchy to a reference network where all the weights are fixed to one. For this purpose, we approximate the two-subunit energy detectors by single linear units whereas the pair of weights associated to each pre-synaptic cell is replaced by a single weight 
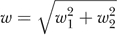
. Using this linearization, we can project unit activations from higher levels back down to the input level, yielding an approximation of their effective RF with respect to the input. Interpreting the resulting activation as a two-dimensional distribution of mass, we compute its normalized inertial tensor *I*. The two eigenvalues of *I* correspond to the two principal axes of the distribution, and therefore yield a measure for the effective RF size. Comparing the optimized to the reference network, we find that the relative difference in the effective RF sizes amounts on average to −23%, −4%, +4%, +7%, and +8% for the five levels, respectively. Thus, while the optimization leads to smaller RF sizes than expected in the lower two levels, the higher levels can increase their effective RFs by converging to nonhomogeneous weight distributions.

**Figure 4:**
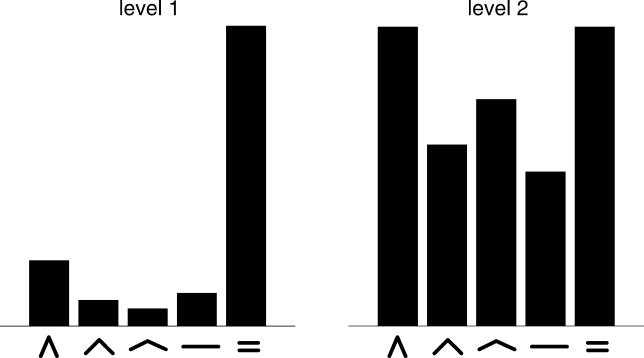
Response Preferences The first two levels of the hierarchy are exposed to five different stimuli, each composed of two bars of equal length in different spatial arrangements (see text). Each stimulus is presented at all possible positions and 12 different orientations within the input space (16 × 16 pixels, the width of the bars is one pixel). All units are assigned to one of the five stimuli for which they respond maximally. The particular position/orientation for which this maximal response is achieved is not considered. The individual distributions of response preferences for the first level units ( *n* = 256) and second level units ( *n* = 128) is shown in the two histograms, respectively.

While early visual areas have been found to preferentially respond to simple oriented gratings, various studies have also reported a selectivity for increasingly complex shapes in subsequent levels of processing [ 22]. In the following we attempt a qualitative comparison with these results, exposing the first two levels of our model hierarchy to five simple stimuli, each composed of two bars of equal length in different spatial arrangements (see Figure 4). For the first four stimuli, the bars are catenated to form an angle of 45°, 90°, 135°, and 180°, respectively. The fifth stimulus consists of two parallel bars. The stimuli are presented at all possible positions and 12 different orientations within the input space. For each unit, the preferred stimulus, for which it responds maximally, is determined. While the units in the first level of the hierarchy show a preference for grating-like parallel bars (68%, Figure 4, left), the selectivity of the units at the second level is more distributed ( Figure 4, right). In particular, the majority of units do prefer stimuli 1–4, in which the two bars are catenated at different angles (74%). This is in accordance with experimental results, which report that cells in higher visual areas such as V2 or V4 do show an increased selectivity for curvature and corner-like shapes [ 22, 23].

**Figure 5:**
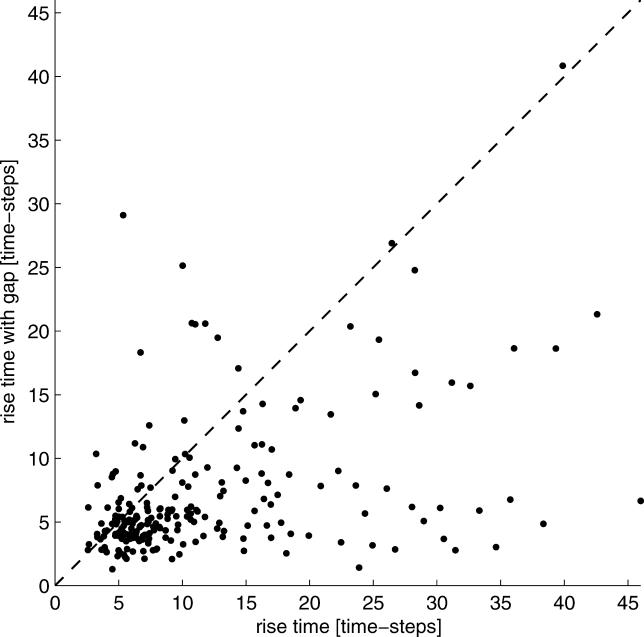
Network Dynamics For each unit at the highest level of the hierarchy, the input frames for which the average response of the unit lies between 25% and 75% of the maximum has been removed from the input stream. Subsequently, the resulting rise time, which is the number of time steps required for a unit to traverse the interval between its 25% and 75% level of maximal response, is plotted versus the rise time under normal input conditions. The ratio “rise time” : “rise time with gap” is 2.1 ± 1.9 (mean ± SD, *n* = 226).

Learning-feature preferences based on a principle of optimal stability run counter to the intuition that biological systems are geared for fast reaction. However, we have to differentiate the behavioral timescale, the stability of visual features, and how fast these are processed by the sensory system. To further elucidate this distinction we investigated the dynamics of cells at the highest level with respect to a modified input stream. Visual stimuli recorded by the moving robot were related to its trajectory and place fields of the cells investigated. We cut the video stream, deleting sections where the robot was moving from an area of low average activity of a considered cell (< 25% of maximum) toward an area of high average activity (> 75% of maximum). The resulting video thus contains sudden jumps from low to high average activity of a particular cell. In Figure 5 we compare the resulting dynamics to the processing of unmodified videos. The scatter plot demonstrates that processing of rapidly changing stimuli by the network is fast, most often a few time steps only. The dynamics of the complete system is dominated by the behavioral timescale, slower by a factor of two. Thus, the system rapidly processes learned optimally stable features.

**Figure 6:**
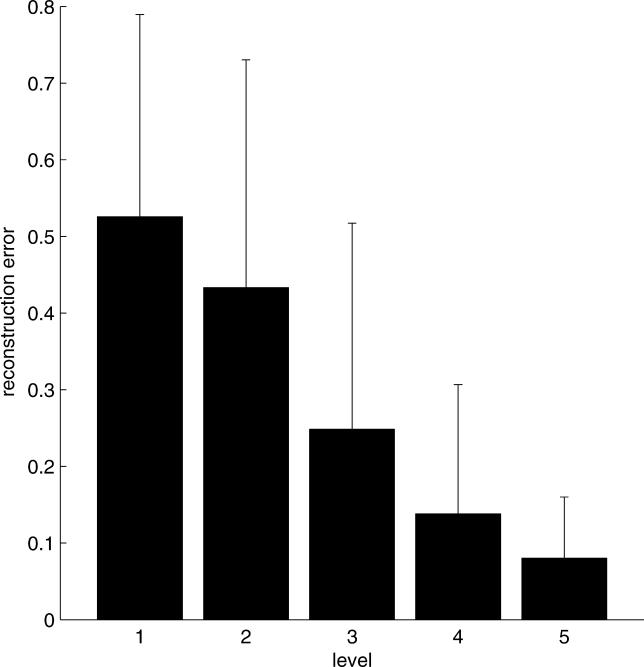
Position Reconstruction The position of the robot is reconstructed using the responses of the *N _l_* = 2 ^9− *l*^ units in the different levels *l* = 1…5 of the processing hierarchy using a standard Bayesian framework (see text). The reconstruction error, defined as the Euclidean distance between the true and the reconstructed position, is shown as a function of the hierarchical level. The error bars indicate the standard deviation from the mean.

To evaluate our hypothesis that the highest level of our model forms place fields, we assessed whether these allow an accurate reconstruction of the position of the robot. We use a standard Bayesian framework for position reconstruction [ 24]. Half of the responses recorded over 10 ^5^ time steps from units at the last hierarchical level serve to acquire the distribution of posterior probabilities *P*( **A**| **x**) where **x** is the position of the robot within the environment, and **A** a vector containing the responses *A _i_* of the individual cells. The other half is used for testing the quality of reconstruction. According to Bayes rule, *P*( **x**| **A**) ∝ *P*( **A**| **x**) **P**( **x**), where **P**( **x**) is the probability of the robot to be at a certain position within the environment. The most likely position of the robot is then given by ◯ = argmax _x_ *P*( **x**| **A**). Applying this procedure to the responses of the different levels in the processing hierarchy yields a monotonically decreasing reconstruction error when moving from lower to higher levels ( Figure 6). In particular we find that responses of the units at the fifth level allow a highly accurate position reconstruction with an average error of 0.08 ± 0.08 (mean ± SD, in units of the length of the long side of the environment). In addition, we analyzed the spatial distribution of reconstruction errors, which shows that reconstruction is good for the central part and becomes poorer around the border of the environment ( Figure S1). Thus, these allocentric representations at the highest level allow a reconstruction of the position of the behaving system with an accuracy equivalent to that observed in reconstructions based on the responses of hippocampal place cells [ 24].

**Figure 7:**
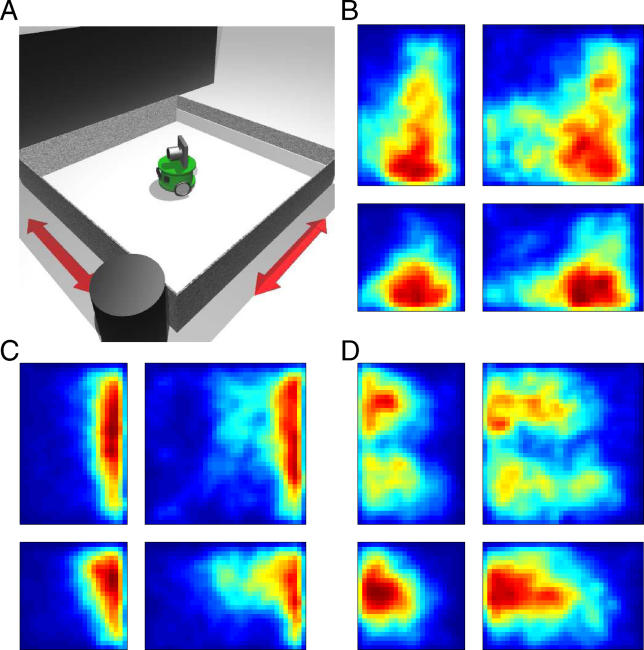
Environmental Manipulations (A) The virtual robot environment consists of a square arena of comparable relative size to the real-world setup and surrounding objects, e.g., a large wall along one side of the arena and a cylinder next to one of the opposite corners. This environment is stretched along either or both directions indicated by the red arrows by a factor of 1.5. (B–D) Response map of three example units that have been acquired in the original environment—small square, lower left map in (B–D)—and subsequently tested in three variants of the original environment, i.e., stretched along the vertical and/or horizontal directions. For each unit, the intensity scale of all four response maps is normalized to the maximal response of all environments.

An important property of place fields in the hippocampus is their response to changes in the environment [ 21, 25]. For instance, it was shown by stretching a rectangular arena along its principle axes that localization and shape of place fields in rat hippocampus are controlled by the distance to the walls and surrounding landmarks [ 26]. As a comparison we perform the same manipulations using a virtual environment ( Figure 7A). After learning in the small square environment, the network connectivity is frozen and exposed to three test environments ( Figure 7B– 7D). We observe that the place fields depend on the robot's distance from one or more of the four surrounding walls in close analogy with the experimental data. Furthermore, three main effects with respect to the stretching of the environment can be distinguished. The place cells either keep a fixed distance to one wall, split into two subfields, or stretch along the direction the environment is stretched. The units shown in Figure 7B and 7C both stretch vertically while maintaining a fixed shape and distance to the right wall. The unit shown in Figure 7C is selective for a certain distance from both top and bottom walls as well as from the left wall, such that the place field is split in the vertical and stretched in the horizontal direction. These results match the properties of neurons observed in the hippocampus and suggest that optimally stable representations capture important aspects of representations in entorhinal cortex and hippocampus.

**Figure 8:**
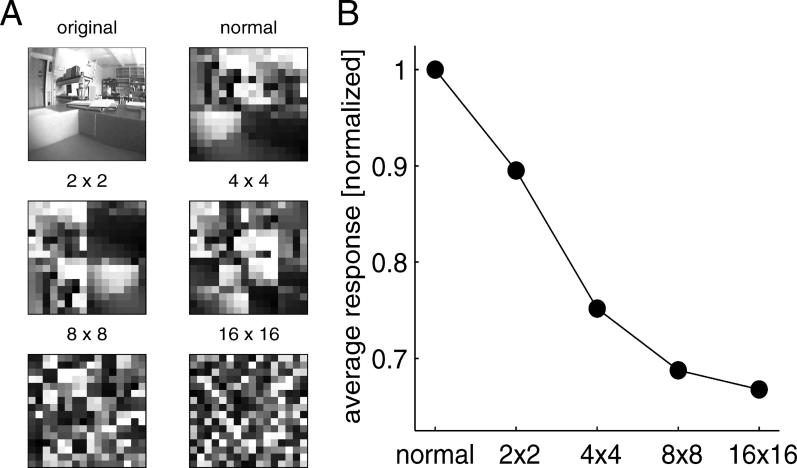
Input Scrambling (A) The original image shows the environment as it is perceived by the camera mounted on top of the robot. The normal image is the downscaled 16 × 16 pixel version that serves as the input to the proposed network model. The four subsequent images show the same input image scrambled by increasing degrees, i.e., by randomly permuting 2 × 2, 4 × 4, 8 × 8, or 16 × 16 blocks of equal size. (B) Average response of the units at the third level of the hierarchy for a normal visual input as well as the four different scales of spatial scrambling.

To assess the properties of cortical representations at higher stages of the visual hierarchy, such as the IT, image scrambling has been successfully used in both experimental [ 27, 28] as well as theoretical [ 12] studies. Here we apply this scrambling method to perform a similar analysis on our intermediate level of the hierarchy, i.e., the “landmark” cells at the third level. Those cells do qualify best for IT-like cells, not only due to their relative position within our visual hierarchy, but also because they show maximal view selectivity ( Figure 3B). We freeze the weights in the network after learning the real-world environment and subsequently perform four different tests with input streams spatially scrambled at four different scales ( Figure 8A). We observe that the average activity decreases monotonically relative to control ( Figure 8B), suggesting that these units are selective for complex visual features characterized at multiple spatial scales. This result is compatible with recent experimental studies [ 27, 28] that have shown a similar characteristic relationship between the spatial scale of scrambling and the degradation of the responses in IT.

## Discussion

We have presented a hierarchical model of an input processing-pathway that is constructed from uniform cortex-like neuronal elements. The local learning rule that modifies the synapses of the feed-forward connections between subsequent levels optimizes the receptive fields to extract smoothly varying features in their afferent input. This model, when exposed to a continuous stream of visual inputs derived from a camera mounted on a mobile robot, develops receptive fields that resemble those observed in the ventral visual pathway. At the highest level of the hierarchy, we observed receptive field properties similar to place fields in the entorhinal cortex and hippocampus. Responses of cells at this level allowed an accurate reconstruction of the position of the robot. Moreover, the model shows a specific change in its response to scrambled stimuli similar to what has been observed in the rhesus monkey.

Every hierarchical neural network model should incorporate nonlinear transfer functions. Otherwise, the hierarchy could in principle be collapsed to one equivalent single layer. However, the choice of such a transfer function is largely unconstrained. Riesenhuber and Poggio [ 12], for instance, proposed the max-function (L ^∞^-norm) to pool over lower-level feature detectors in order to gain invariances while maintaining feature specificity. Here, in contrast, we have used a saturating energy detector, which in the nonsaturating region corresponds to an L ^2^-norm. This particular choice is inspired by a previous study, where not only the feed-forward connections but also the degree of nonlinearity was subject to the optimization procedure [ 29]. In accordance with experimental findings [ 30], this theoretical study has shown that most units converge toward an L ^2^-norm. In addition, it has been reported that this choice of nonlinearity combined with optimization for temporal stability leads to the formation of units that are optimally invariant while highly feature selective [ 20].

One of the appealing aspects of using objective functions to model cortical architectures is that they propose a small set of computational principles underlying cortical and subcortical processing in the visual and auditory system [ 16]. As such, this approach facilitates the investigation of the basic question of how the relatively uniform anatomical structure of the cerebral cortex can generate a highly diverse set of functional and physiological properties [ 31, 32]. Many years back, Lashley tried to capture this issue with his concept of equipotentiality [ 33]. Although the original interpretation of Lashley is highly controversial [ 34], it does highlight that the computational principles underlying cortical circuits are at least partially modality- and area-independent [ 35, 36]. For instance, it has been shown that routing projections from the retina to the auditory pathway leads to the development of cells in the auditory cortex with properties similar to those found in primary visual cortex [ 37]. Similarly, an fMRI study with blind human subjects has shown that cortical regions that are normally involved in processing visual information are activated verbal-memory tasks as well as braille reading [ 38]. While these results suggest a generic computational architecture across modalities, it is unclear whether the same holds for different levels of processing within one modality. The model proposed here shows that generic computational principles, temporal stability, and local memory, can underlie the generation of different levels of processing within one modality and that the variability in functional organization can be accounted for in terms of the statistics of the inputs each level is exposed to.

## Materials and Methods

### Experimental setup

We performed the real-world experiments using the Khepera robot K-Team, Lausanne, Switzerland ( Figure 1A). The simulated agent was implemented in C++ using the Open Graphics Library. The robots randomly explore an environment that consists of a rectangular arena confined by walls and surrounding objects/cues. For the real-world robot, these cues are present in the office environment within which the experiments are performed. For the simulated robot, the cues are well-defined objects, i.e., a black wall and a black cylinder ( Figure 7A). The environments are explored using a random sequence of translations (maximum 0.25 environment lengths/s) and rotations (maximum 90 °/s) combined with obstacle avoidance at the walls. At each point in time, the robot switches its behavior from translation to rotation or vice versa with a probability of 0.1. As soon as an obstacle is detected by the infrared sensors arranged around the cylindrical body of the robot ( Figure 1A), the robot turns away until the obstacle is no longer sensed. A camera with a view-angle of 100° (120° for the virtual environment) provides the visual stimulus of 16 × 16 pixels. This image is passed through edge-detection before it is presented to the network model described next. The position as well as the orientation of the real-world robot was tracked using a second CCD camera mounted above the arena.

### Network

The network consists of a hierarchy of five levels with intralevel connections and purely feed-forward processing between levels. Each level *l* = 1...5 is represented by a lattice of *N _l_* = 2 ^9− *l*^ identical computational units. Each unit comprises a two-subunit energy detector [ 20, 29, 39]. The activity of a unit *i* at time *t* and level *l* is given by



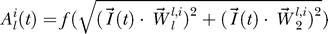



where *f*( *x*) = 1 − *e* ^− *x*^2^^ is the unit's nonlinear, saturating activation function. The weight vectors 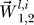 characterize the linear feed-forward mapping of the two subunits, respectively. The vector 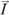
( *t*) represents the main input to the hierarchy ( *l* = 1) or the output 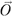
_*l*−1 _( *t*) of the afferent level ( *l* > 1). The latter is computed from the level's activity according to the following equation:



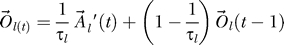



where



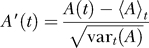



〈·〉 *_t_* is the temporal average and var *_t_*(·) the variance over time. Thus, the output is a running average of the activity, mean-corrected and normalized to unit variance. This leaky integration over time, with a time-constant of *τ _l_* = 2 *^l^* time steps, constitutes the local memory of each unit.

The feed-forward connectivity between the levels of the hierarchy are chosen such that the relative arbor within the afferent level increases while moving up the hierarchy. For this purpose, the units in the different levels are arranged in three three-dimensional lattices. All units in a level then receive input from a subset of units of equal size, geometrically aligned with respect to the first two dimensions of the lattices such that an even coverage of the afferent level is achieved (see Table 1).

**Table 1:**
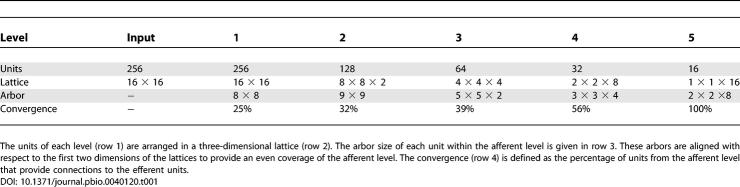
Feed-Forward Network Connectivity

In contrast to the feed-forward mapping between levels, the intralevel connections do not directly influence a unit's activity but merely exchange learning signals between units. These learning signals serve to decorrelate the representations formed within a level and are directly derived from the objective function described below.

### Optimization

The system is using an online learning algorithm as opposed to batch learning and therefore all the statistics are computed continuously using running averages, with a characteristic time-constant of a 1000 time steps. The weight vectors 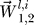 are subject to unsupervised learning which aims to maximize the objective function *ψ _l_* for each level, using standard gradient ascent.



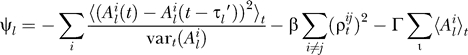



where 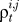 is the temporal correlation between units *i* and *j,* i.e.,



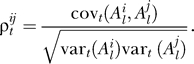



cov *_t_* (·,·) is the covariance over time.

The first term of the objective function becomes small for signals varying smoothly/slowly over time with respect to the timescale given by 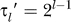 time steps. In order to prevent the trivial solution 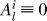, this term incorporates a division by the unit's variance. Minimizing the second term forces pairs of units to become maximally decorrelated. Please note that in contrast to the first term of the objective, which solely incorporates information local to each unit 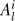, the decorrelation term requires information from two units 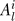 and 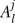 for *i* ≠ *j*. This information is exchanged through the lateral connectivity within a level, whereas its extent determines which pairs of units are decorrelated ( Figure 1B). In our experiments, we chose to decorrelate all pairs of units that share common feed-forward input. The last term implements a form of regularization that aims to reduce the average activity of each unit. The relative importance of the three terms is controlled by the parameters *β*, *Γ* ≥ 0. For *β* ≪ 1, the first term dominates such that the units' activity become maximally stable while being strongly correlated. For *β* ≫ 1, the units' activities become well-decorrelated but fail to extract the stable features from their input. Thus, *β* must be chosen between these extreme cases to allow an optimal balance between the two first terms of the objective. The particular choice for *Γ* was found to be less critical. For all the experiments presented in this study, we used *β* = 5/ *N _l_* and *Γ* = 20/ *N _l_* where *N _l_* is the number of units in level *l*.

## Supporting Information

Figure S1 Spatial Distribution of Reconstruction Error The position of the robot is reconstructed based on the responses from the 16 units at the highest level of the hierarchy. The resulting reconstruction error is color-coded as a function of the position within the environment. The reconstruction quality is good in large parts of the central region of the environment and becomes poorer at the borders. The latter is due to two issues: 1) the extreme border regions of the environment are not visited as often such that the estimation of the posterior probabilities becomes less accurate leading to large errors; 2) when the robot faces the wall around the border of the environment, its visual stimulus is dominated by the wall, which looks identical from different positions. This leads to perceptual singularities (i.e., same perception for different locations), yielding similar network activation patterns which can result in large reconstruction errors.
(2 KB PDF)

## Abbreviations

IT: inferotemporal cortex
RF: receptive field
