# Supplementary material for: A Model of the Ventral Visual System Based on Temporal Stability and Local Memory
Source: PLoS Biol. 2006 Apr 18;4(5):e120. doi: 10.1371/journal.pbio.0040120 (PMC1436026; doi:10.1371/journal.pbio.0040120)
Supplement: Supplementary file 1 [file pbio.0040120.sg001.pdf]

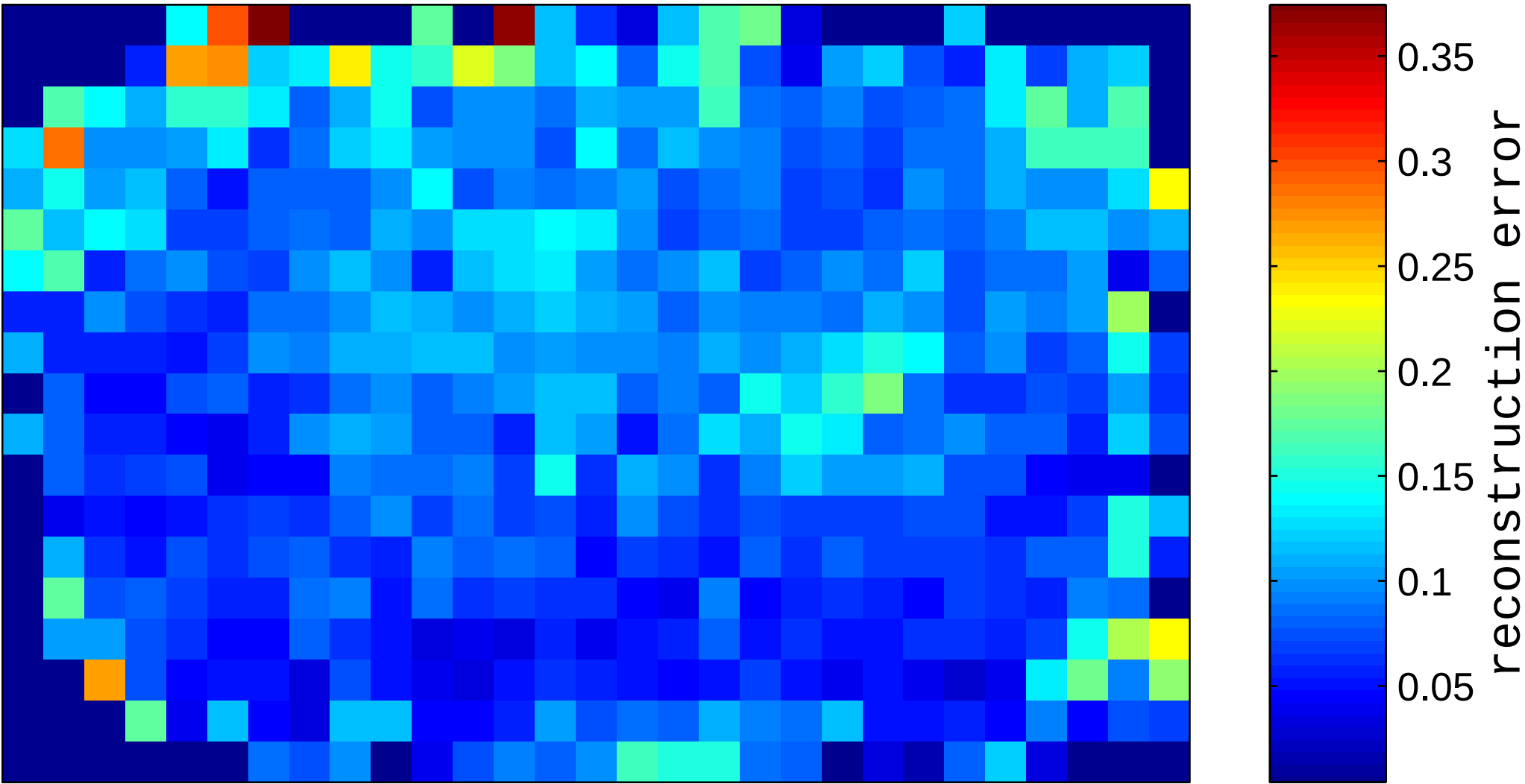
0.35
0.3
0.25
0.2
0.15
0.1
0.05
reconstruction error
